# From time-series transcriptomics to gene regulatory networks: A review on inference methods

**DOI:** 10.1371/journal.pcbi.1011254

**Published:** 2023-08-10

**Authors:** Malvina Marku, Vera Pancaldi

**Affiliations:** 1 CRCT, Université de Toulouse, Inserm, CNRS, Université Toulouse III-Paul Sabatier, Centre de Recherches en Cancérologie de Toulouse, Toulouse, France; 2 Barcelona Supercomputing Center, Barcelona, Spain; University of California Irvine, UNITED STATES

## Abstract

Inference of gene regulatory networks has been an active area of research for around 20 years, leading to the development of sophisticated inference algorithms based on a variety of assumptions and approaches. With the ever increasing demand for more accurate and powerful models, the inference problem remains of broad scientific interest. The abstract representation of biological systems through gene regulatory networks represents a powerful method to study such systems, encoding different amounts and types of information. In this review, we summarize the different types of inference algorithms specifically based on time-series transcriptomics, giving an overview of the main applications of gene regulatory networks in computational biology. This review is intended to give an updated reference of regulatory networks inference tools to biologists and researchers new to the topic and guide them in selecting the appropriate inference method that best fits their questions, aims, and experimental data.

## 1. Introduction

**Fig 1:**
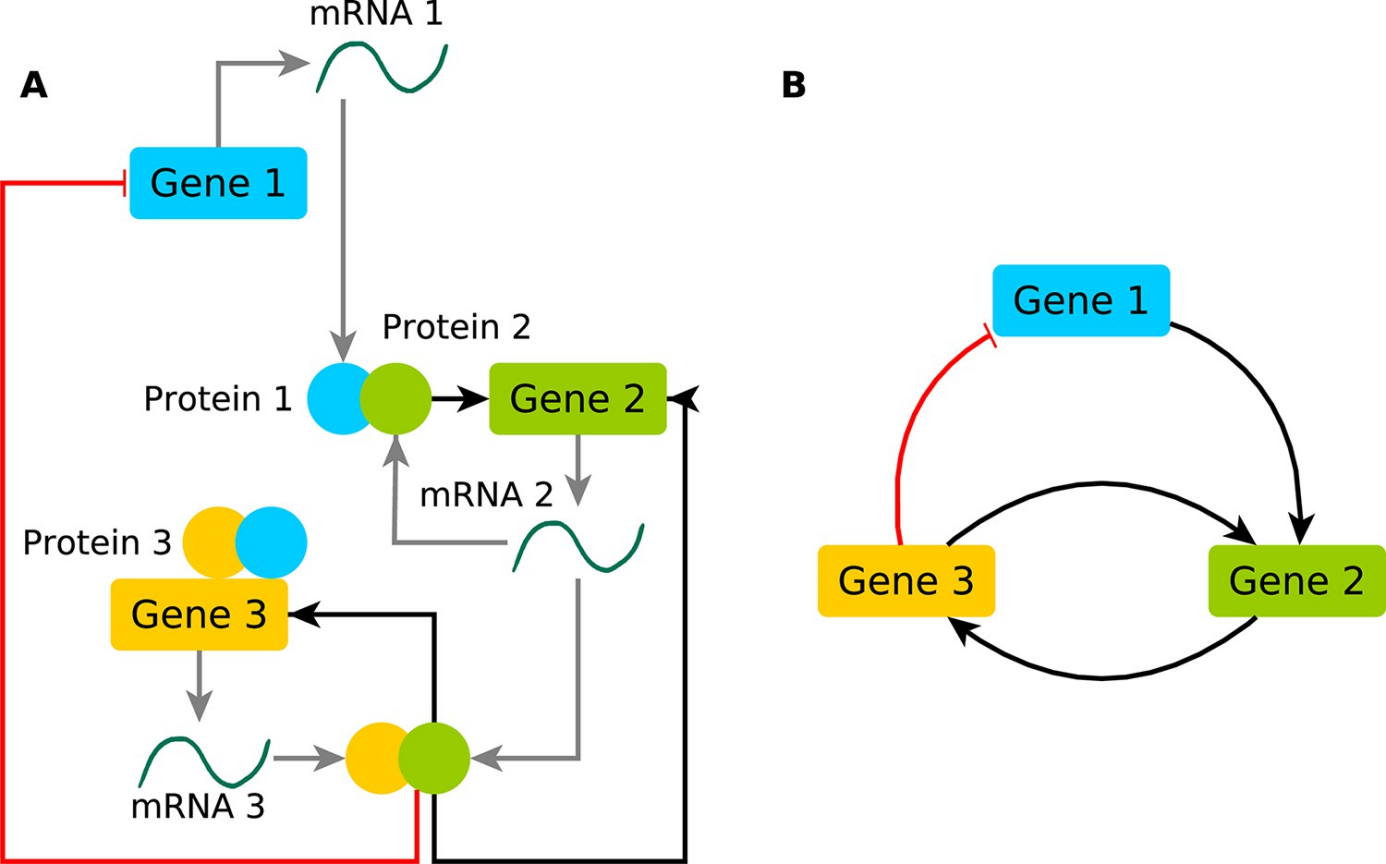
**(A)** A toy model of gene regulation of 3 genes involved in a transcriptional regulatory network, showing genes transcribed into mRNAs and translated into proteins that regulate another one of the genes. **(B)** Compressed representation of the interactions on the left as a GRN in which only genes are shown with their regulatory interactions.

In complex system theory, a system is defined as complex if certain properties, such as nonlinearity, feedback loops, adaptation, and nontrivial behavior, emerge from the collective interactions between the system components and the surrounding environment [1]. As such, all biological systems, especially molecular systems, are inherently complex, and the global structure and behavior of the system cannot be straightforwardly inferred from the (local) properties of its components. For example, characterizing the connection between genotype and phenotype and, furthermore, pathology, requires not only the identification of the molecules involved in the process and their specific characteristics but also a description of the ways in which these molecules interact with each other across spatiotemporal scales. To facilitate the representation and study of such complex systems, their interacting components can be represented as a network, commonly visualized as a graph of *nodes* (*vertices*) connected by *edges* (*links*) (Fig 1). In a molecular network, the nodes represent molecular objects of interest (e.g. genes, mRNAs, transcription factors (TFs), whereas the edges represent the interactions between them (e.g. protein binding, gene co-expression, TF–target regulation, etc.). Therefore, depending on the types of nodes and edges considered, different molecular networks exist, like protein–protein networks (PPI) [2–5], gene regulatory networks (hereafter, GRNs) [6,7], signal transduction networks [8,9], etc. Strictly speaking, we define GRNs as networks including any type of regulatory interactions between regulatory and target molecular entities (miRNAs-targets, RBP-targets, kinases/phosphatases-substrates). Here we will focus mostly on GRNs that describe interactions between molecular components at the transcriptomics level, thus including TFs and their targets.

In studying GRNs, 2 main approaches for extracting information exist: (i) *static network analysis*, and (ii) *dynamical modeling*, each of which offers different amounts and types of information regarding the network organization, topology, and behavior [10,11]. Inferring and modeling these regulatory networks, however, is a challenging reverse engineering process and requires the combination of both a thorough biological understanding of the system, as well as accurate and advanced computational inference methods [12]. Moreover, the advanced technological improvements in measuring gene expression, in cellular populations or even single cells, and the increasing interest in clinical applications of genomics, confer importance and relevance to data-driven GRN inference methods. These approaches can ultimately provide considerably more insights on gene regulation mechanisms, drugs’ mode of action, pathway perturbation, etc. than the original data alone.

With the continuous advances in biotechnology, inference methods have also evolved from using bulk gene expression data [13–16] to single-cell transcriptomics [17–22]. Additionally, some methods were adapted to infer the regulatory networks from time-series and/or pseudotemporal single-cell transcriptomics, in which case more accurate knowledge on gene–gene interaction can be inferred [20,23,24].

Consequently, highly performing algorithms have been developed over the years, with increasing accuracy, robustness, and applicability, and incorporating additional computational analyses, such as filtering for the presence of TF motifs in promoters of their inferred targets [25,26]. It is important to note, however, that GRN inference is not the final aim. Our goal in performing such calculations is to produce insight on the biological processes considered, aiming to uncover new functionally important molecular regulatory interactions or propose new drug targets [6]. Additionally, network inference is a prerequisite to applying dynamical models to GRNs, in order to increase the models’ power to understand and predict the system’s temporal behavior [12].

Inference of data-driven accurate and powerful GRNs remains an open and evolving computational challenge, and new inference methods are being published continuously. In this review, we give a description of GRNs and describe the algorithms behind a selection of the available methods on network inference from experimental data. Several reviews on GRN inference based on different data sources have been published [27–30], many of them reviewing and performing benchmarking analysis on their performance based on simulated or real case datasets [31–34]. While almost all of them consider methods based on steady-state bulk or single-cell datasets, here, we will focus particularly on pseudo/time-series transcriptomics-based inference algorithms, including some methods exploiting the pseudotemporal formalism applied in single-cell datasets, but mostly focusing on inference based on bulk RNAseq time courses. We aim to give researchers a roadmap from the simplification of biological processes in the form of regulatory networks, to an algorithmic review of GRN inference methods based on time-series transcriptomics. Our goal is to guide researchers, but the choice of a method will ultimately depend on the scope and purpose of their research. Furthermore, we list some of the recent applications of GRN inference methods in various fields, particularly in cancer research, and give an introduction to dynamical modeling of GRNs, as a promising tool to exploit GRNs in the generation of new biological hypotheses. We conclude by discussing some pitfalls and existing challenges in data-driven GRN inference methods and speculate on possible directions for future developments in this field.

## 2. Gene maps: Network representation of gene regulation

Although a single definition does not exist, we define GRNs as topological maps representing relationships and interactions between biological entities. Interactions between proteins, TFs, and genes can be represented as directed graphs of *nodes* and *edges*. Notably, the directionality of the edges (defining the source and the target in the interaction) is not present in all biological networks, but it is an important feature in the case of the regulatory networks or metabolic networks, defining the direction of information flow. In these networks, additional information is added by indicating specific types of interactions, represented by signed edges (Fig 1B). In the simplest case, interactions are categorized as *activations* and *inhibitions*, represented as positive and negative edges accordingly. GRNs are composed of regulatory nodes (source/cause nodes) and regulated nodes (target/effect nodes), generally mapped as TF–target gene networks, with the incoming connectivity (in-degree) estimated using gene-centered approaches, and the outgoing connectivity (out-degree) using TF-centered approaches.

The structure of the network enables the calculation of various quantities that capture different features of the network topology that can reveal important information on the underlying biology of the system. Centrality measures, such as eigenvector centrality, Page/Chei Rank, Burt’s constraint, or alpha centrality, have been shown to highlight key nodes in a network based on connectivity and influence [35–38]. Particularly of interest, in combination with mathematical methods such as linear approximation, Signal Flow Analysis, and Feedback Vertex Set Control [39], the network topology can give valuable information on the determinant nodes that define the system’s temporal behavior [40]. It is important to note that interpreting the topological results in GRNs requires careful consideration of other implicit factors linked to the biological context of the system under study. For example, the role of a gene in a GRN and its regulatory interactions can depend on the cellular or environmental conditions in which it is expressed. These context-dependent factors are not captured by the centrality measures, resulting in incomplete descriptions of gene regulation. More on these open challenges will be discussed in Section 8.

## 3. Inference methods

By definition, the process of inferring the network structure of the system based on experimental data is a reverse engineering process, usually referred to as GRN inference. In general, depending on the input data used, GRN inference methods can be categorized into 2 types: (i) *steady state gene expression*, and (ii) *time-series gene expression* inference methods. In the first category, in principle, the GRN inference is obtained by considering perturbations or instances of the system and estimating the gene expression after it reaches an equilibrium. In the second category, the input data consist of gene expression measured at several time points after a perturbation, thus providing a temporal evolution of the transcriptome. Consequently, time series can be more informative than static data in a wide range of situations to infer gene functionalities, interactions, and causal relationships, as well as to establish potential clinical implications of gene expression dynamics determined by these relationships [41]. Both methods, however, have various limitations, mostly stemming from technical issues that arise from the experimental protocols: sampling time points, cost, cell synchronization, sparsity of gene expression data, etc. Therefore, several computational methods that combine both steady-state and time-series approaches have been developed, usually based on advanced machine learning algorithms. Additionally, new technologies providing expression at the single-cell level have led to the development of inference methods that are specifically adapted to single-cell transcriptomics [42].

Inferring the functional relationships between genes requires the estimation of gene functional dependencies, which can be broadly achieved by 2 different reverse engineering approaches for GRN inference:

1. ***Model-free methods***: In this approach, gene dependencies are inferred using several statistical and machine learning methods, such as mutual information [43–45], random forest [46–49], or network deconvolution [50,51].
2. ***Model-based methods***: In this approach, a quantitative dynamical model (for example, ODE [24,52,53]), regression method [54], or Bayesian reasoning [55] is defined to model the dynamical properties of the system, while the regulatory network is inferred by optimization of the model parameters based on the time-series data. In this way, model-based GRN methods can reveal some dynamical features of the system, increasing the model interpretability, especially from a biological point of view.

Depending on the scope and biological question for which the GRN is required, model-free or model-based inference methods can be applied. It is important to note that model-based methods are considerably more computationally intensive and might be limited by the underlying nonrealistic linear models of gene expression dynamics. On the other hand, the *Dialogue on Reverse Engineering Assessment and Methods* (DREAM) project, an initiative to benchmark multiple inference methods, indicated that no single method can perform the best in every possible dataset and across different settings. Instead, a high-confidence consensus network, inferred from different methods in a process referred to as Wisdom of Crowds [14], is the most accurate, while also providing an estimation of robustness and performance. For this reason, instead of using a single inference method, other researchers have been developing computational tools combining several GRN inference methods [56], providing a ranking of the methods according to their performance.

The increasing demand for improving the inference accuracy in real datasets has warranted the adaptation of some steady-state inference methods to consider time-series data. This led to the development of new promising approaches, whose applications can generate novel results in various molecular systems. In the next sections, we will elaborate on this category of inference tools specifically and give an overview of several different algorithms and their performance.

### 3.1 Inference from time-series

**Fig 2:**
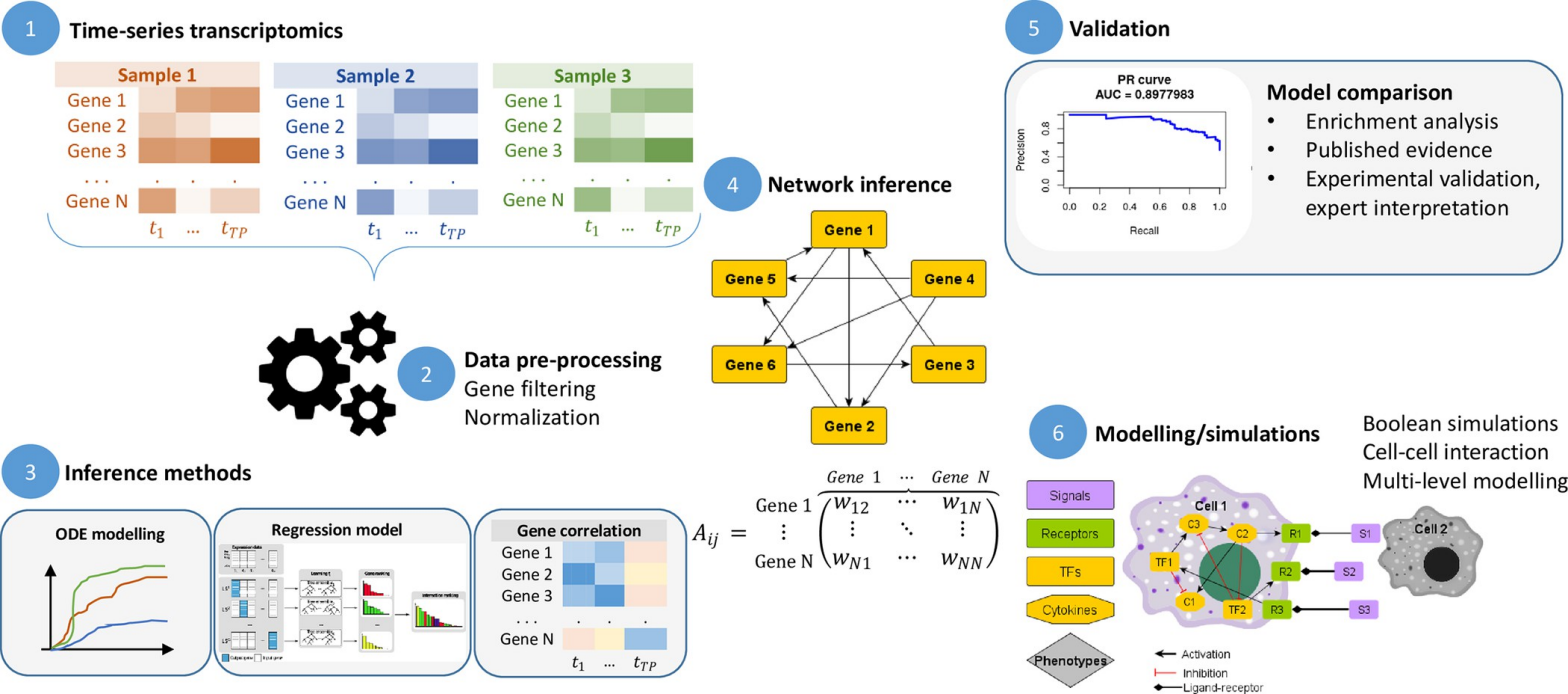
From time-series transcriptomics to GRN inference and model validation. The process usually starts with processing the time-series datasets and identifying the time-points that are relevant to describe the biological process (1). As a second step, data formatting, normalization and gene filtering and/or binarization might be necessary (2), depending on the inference method to be used (3). The inferred network consists of a weighted directed GRN (4). Model validation (5) is then necessary to quantify the method performance in inferring the GRN when compared to gold-standard datasets, evidence from literature or prior biological knowledge. The inferred network can further be used for the application of dynamical models in cellular or multilevel modeling (6).

Inference of gene networks from time-series data provides a more complete picture of the system than using just steady-state data, as the biological system is intrinsically complex and biological function relies on coordinated sets of genes whose expression evolves over time. In general, inference methods based on time-series transcriptomics use a dataset containing a list of genes with their expression measured at several time points. Let us define the dataset *D*_*TS*_, as a matrix with dimensions *N*×*P*, where *N* is the number of genes and *P* is the number of time points at which their expression is measured:

$$D_{TS}=X(t_{1}),X(t_{2}),\ldots ,X(t_{p})$$ (1)

where **X**(*t*_*p*_), *p* = 1,2,…,*P* is a vector of *N* genes with their expression at time *t*_*p*_. The main goal of inference methods is to assign a weight *w*_*j*,*i*_≥0,*i*,*j* = 1,2,…,*N* to any putative interaction between gene *i* (target) and *j* (source), representing a regulatory interaction in the biological system. To this end, different inference methods use various regression tools to model the expression of a gene as a function of its regulators. It is important to note that, usually, the time-series inference methods combine time-series with steady-state datasets, in order to increase the accuracy and predictive power of the inferred network. Independent of the method chosen, the goal is to reconstruct the GRN that would produce the observed profile of expression across time, in the form of a directed graph, in which each edge is associated with its characteristic weight (Fig 2).

### 3.2 Inference algorithms

Based on the inference model they use, inference methods can be grouped into 7 categories, namely, (i) *mutual information (MI)*; (ii) *dynamical Bayesian*; (iii) *Granger causality*; (iv) *Boolean*; (v) *ordinary differential equation (ODE)*; (vi) *graphical Gaussian*; and (vii) *regression*. It is important to note, however, that many methods apply a combination of different models and novel approaches. In the following sections, we will introduce the basic concept of each inference algorithm, while some of the available tools for using each of the methods are summarized in Table 1.

**Table 1:** GRN inference tools from time-series transcriptomics categorized by their inferring algorithm. The characteristics of the inferred network are indicated as follows: ⊘ undirected; ⊳ directed and unsigned; ▶ directed and signed.

Tool	Method	Language	Reference
▶Time-delayed ARACNE	Mutual Information	R	[57]
⊘ MRNET	Mutual Information	minet R package	[58]
⊳ CLR	Mutual Information	minet R package	[43]
⊳ Jump3	Regression	MATLAB	[79]
⊳ dynGENIE3	Regression (+ ODE)	R	[24]
⊳ SWING-RF	Granger causality + Regression	Python	[65]
⊳ BETS	Granger causality	Python	[64]
⊳ CGC-2SPR	Granger causality	R/MATLAB	[66]
⊳ scanBMA	Dynamical Bayesian Model	R	[55]
⊳ Package GeneNet	Dynamical Bayesian Model	R	[81]
⊳ Package G1DBN	Dynamical Bayesian Model	R	[82]
⊳ PROB	Dynamical Bayesian Model	MATLAB	[83]
⊳ MERLIN	Dynamical Bayesian Model	C	[84]
⊳ REVEAL	Boolean Network + MI	C	[70]
⊳ BoolNet	Boolean Network	R	[73]
⊳ ATEN	Boolean Network + Regression	R	[74]
▶ TSNI	ODE	MATLAB	[77]
▶ Inferelator	ODE	R	[76]
▶ DryNetMC	ODE	R	[85]
⊳ BINGO	Gaussian process	MATLAB	[53]

### Mutual information–based models

In these methods, the putative interaction between 2 genes is inferred by estimating the mutual information (MI) between the expression *X*_*i*_ of gene *i* at time point *t*_*p*_ and the the expressions *X*_*j*_ of gene *j* at the *m*^*th*^ previous time points:

$$I_{m}(g_{i},g_{j})=\sum_{p=m+1}^{P}P(X_{i}(t_{p}),X_{j}(t_{p}-m))\log\frac{P(X_{i}(t_{p})X_{j}(t_{p}-m))}{P(X_{i}(t_{p}))P(X_{j}(t_{p}-m))}$$ (2)

where *P*(*X*_*i*_(*t*_*p*_),*X*_*j*_(*t*_*p*_−*m*)) is the joint probability of observing gene *i* at time point *t*_*p*_ with expression *X*_*i*_(*t*_*p*_) and the expression *X*_*j*_(*t*_*p*_−*m*) of gene *j* at time point *t*_*p*_−*m*, whereas *P*(*X*_*i*_(*t*_*p*_)) and *P*(*X*_*j*_(*t*_*p*_−*m*)) are the marginal probabilities of observing gene *i* at time point *t*_*p*_ with expression *X*_*i*_(*t*_*p*_) and the expression *X*_*j*_(*t*_*p*_−*m*) of gene *j* at time point *t*_*p*_−*m* independently. Estimating *I*_*m*_(*g*_*i*_, *g*_*j*_) for each pair of genes and each time point, an interaction *g*_*j*_→*g*_*i*_ is being considered if *I*_*m*_(*g*_*i*_, *g*_*j*_) exceeds a defined threshold. Among the methods using MI algorithm for GRN inference, we can highlight TimeDelay-ARACNE [57], MRNET [45,58], and CLR [43]. One of the main advantages of these methods lies in their simplicity and speed of computation. On the other hand, their main limitation is that they do not give information on the nature of inferred interactions (activation or inhibition).

### Dynamical Bayesian models

Dynamical Bayesian network (DBN) model GRN inference methods from time-series datasets [59,60] come as an extension of simple Bayesian network (BN) models applied to steady-state datasets, in order to overcome the limitation of prohibiting the existence of feedback loops in the network. Given a GRN defined as a set of vertices and edges *G* = (*V*,*E*), the method searches for any relationship between the expression of a target gene (defined as a random variable) and its regulators (defined as parent genes) by calculating a joint probability distribution. In a time-series experiment, consisting of *N* genes and *P* time points, the joint probability distribution of each gene is defined as:

$$P(X_{1}(t_{1}),X_{1}(t_{2}),\ldots ,X_{1}(t_{P}),\ldots ,X_{N}(t_{1}),X_{N}(t_{2}),\ldots ,X_{N}(t_{P}))=P(X(t_{1}))P(X(t_{2})|X(t_{1}))\ldots P(X(t_{P})|P(X(t_{P-1}))$$ (3)

where **X**(*t*_*p*_), *p* = 1,2,…,*P* is a vector representing the gene expression values at time point *t*_*p*_. The network *G* is then inferred by identifying the network structure with the highest posterior probability of each edge *E* from the data, assuming a linear dependency between previous and current gene expression. The network *G* is Markovian, meaning that each gene is dependent only on the regulation by its regulators (parents). An edge is then included in the network if the marginal posterior probability of an observation (left hand side of Eq 3 when written for each gene) exceeds a given threshold. Despite having a generally good performance when benchmarked against other time-series GRN inference methods (DREAM5 challenge [14]), DBNs are generally computationally expensive and usually limited to small size networks. To overcome this limit, several methods like CAS [61] or scanBMA [55], implemented as a function in networkBMA R package [62], have been developed, combining DBN with machine learning methods or prior knowledge to guide the search for the regulators of each gene.

### Granger causality models

In these methods, 2 assumptions in defining the expression *X*_*i*_ of a gene *i* are made: (1) the expression *X*_*i*_ of gene *i* at time point *t*_*p*_ is a function of its expressions at the *m* previous time points; (2) the expression *X*_*i*_ of gene *i* at time point *t*_*p*_ is a function of its expressions at the *m* previous time points, combined with the expressions *X*_*j*_ of gene *j* at the *m* previous time points, thus indicating the effect of gene *j* on gene *i*. If the second assumption is significantly more successful than the first assumption, it is said that gene *j* Granger causes gene *i*.


$$X_{i}(t_{p})=\alpha_{0}+\alpha_{1}X_{i}(t_{p}-1)+\ldots +\alpha_{m}X_{i}(t_{p}-m)+\eta (t_{p})$$ (4A)

$$X_{i}(t_{p})=\alpha_{0}+\alpha_{1}X_{i}(t_{p}-1)+\ldots +\alpha_{m}X_{i}(t_{p}-m)+\beta_{0}+\beta_{1}X_{j}(t_{p}-1)+\ldots +\beta_{m}X_{j}(t_{p}-m)+\eta (t_{p})i,j=1,2,\ldots ,N\;p=1,2,\ldots ,P$$ (4B)

where *α*, *β* are coefficients and *η*(*t*_*p*_) is the residual noise at time *t*_*p*_. Generalizing for *N* genes, gene *j* (*j* = 1,2,…,*N*,*j*≠*i*) is said to be causal for gene *i* if considering the expression of gene *j* in the previous time points significantly improves the prediction of the expression of gene *i* at the current time point. Notably, when referring to the “previous time points,” several previous time points (*t*_*p*_−1, *t*_*p*_−2, etc.) can be considered. Considering *N* genes in the dataset, the vector autoregressive (VAR) model is used to estimate the Granger causality over the entire list of genes, where a linear dependency between genes is being assumed [63]. Several methods using Granger causality for GRN network inference have been developed, such as BETS [64], SWING [65], CGC-2SPR [66], among others. One of the notable advantages of this approach is the computational efficiency and speed, compared to other inference methods, with a similar performance. However, due to the definition and underlying algorithm, Granger causality inference methods can be used only on time-series datasets, which often suffer from sparsity and nonuniformity of time-points. For this reason, a flexible time lag between consecutive time-points can be applied as suggested in [65].

### Boolean models

In these methods, a GRN is represented as a graph *G* = (*V*,*E*) and <*i*,*j*,*s*> indexes, in which *i* represents the target and *j* represents the source gene. Each edge is characterized by a sign *s*∈{+,−} indicating the type of interaction between the regulator and the source node. Generally, a positive sign indicates a positive (activating, promoting) relationship between a regulator and its target gene (i.e., the regulator gene *j* contributes in increasing the expression of target gene *i* through specific biological processes), whereas a negative sign indicates a negative (inhibiting, repression) relationship (i.e., the more expressed the regulator gene *j* is, the less expressed the target gene *i* will be). Notably, the expression of each gene is given in Boolean (binary) values {0,1}, thus providing only a qualitative description of gene regulation and expression. Given a time-series dataset, the expression *X*_*i*_ of gene *i* at time *t*_*p*_ is given by a Boolean function of its regulators:

$$X_{i}(t_{p})=F_{B_{i}}(X_{i,1}(t_{p}-1),X_{i,2}(t_{p}-1),\ldots ,X_{i,k}(t_{p}-1))$$ (5)

where *X*_*i*,*k*_, *k* = 1,2,…,*N*−1, *k*≠*i* are the regulators of gene *i*, and $F_{B_{i}}$ is a Boolean function describing the regulation of gene *i* by using the Boolean operators AND, OR, and NOT. In this way, the inference of any causal relationship is performed through a qualitative dynamical modeling of gene expression [67,68]. Compared to the other inference methods, BN inference methods offer the advantage of being parameter free and relatively easy to apply. On the other hand, they suffer from several drawbacks and limitations, arising from the discretization/binarization process and the finite space explored for inferring the regulatory processes (limited number of possible BNs), and the qualitative description of expression [69]. Nevertheless, several inference methods based on BN have been developed, such as REVEAL [70], Best-Fit [71,72], both available as extensions in the BoolNet R library [73], and ATEN [74].

### Ordinary differential equation (ODE) models

In this formalism, the variation of expression *X*_*i*_ of gene *i* at time *t*_*p*_ is given by a nonlinear function of its regulators (including self-regulation) at the same time steps:

$$\frac{dX_{i}}{dt}=\sum_{k=1,k\neq i}^{N-1}f(X_{i,k})+\eta_{i}$$ (6)

where the functions *f*(*X*_*i*,*k*_) of *k* regulators of gene *i* with expression *X*_*i*,*k*_ are usually assumed as polynomial functions [75], and *η*_*i*_ represents the nondeterministic term (noise) in the regulatory function of *X*_*i*_. In this way, the regulatory network is inferred by solving a system of ODEs (one equation for each gene). From its definition, solving the system of ODEs Eq 6 is computationally and conceptually challenging, even in a linear space, as these systems are defined by a large number of interacting coefficients, for which the prior information is limited or missing. Consequently, ODE methods are limited to network inference on a small number of genes and are usually combined with other inference methods in order to reduce the computational complexity [44,45]. In principle, a causal relationship between 2 genes is considered if the interaction is described by a relatively high interacting coefficient. Despite the difficulty of applying them to real datasets, several methods using ODE models have been developed, including Inferelator [76] and TSNI [77], but they often display a relatively lower performance than alternative approaches, as reported in [64].

### Regression models

Generally, in these methods, a nonlinear regression or ODE formulation is used to model the expression of a gene at a certain time point/condition as a nonparametric function of the expression of other genes at the same time point/condition:

$$X_{i}(t_{p})=f_{i}(X_{j\neq j}(t_{p}))+\eta (t_{p})$$ (7)

where *η*(*t*_*p*_) is a random noise for the $t_{p}^{th}$ time point. The inference of *f*_*i*_ functions is then performed by implementing a feature selection algorithm, like LASSO, least angle regression (LARS), or random forest algorithm, to “learn these functions from an ensemble of regression trees” [78]. Identifying the candidate genes on the regulation of *N* genes starts by splitting the problem into *N* distinct subproblems, thus assuming that each gene can be a target gene and a potential regulator. Causal interactions are quantified by estimating the confidence levels of each *j*→*i* interaction, representing the weights *w*_*j*,*i*_. Importantly, in order to increase the accuracy of the inferred network, some tree-based methods have been adapted to use both time-series and steady-state datasets. Some inference methods based on decision-tree algorithms can be highlighted for having particularly good performance: dynGENIE3 [24] and Jump3 [79], both being extensions of GENIE3 [46] on time-series datasets.

### Gaussian process (GP) models

In these methods, a GP regression is used to model the relationship between the current expression of the genes and their previous expression levels, with the functional relationships between targets and their regulators modeled as Gaussian processes:

$$X_{i}(t_{p})=f(X_{j}(t_{p}))+\eta (t).$$ (8)

A causal relationship (edge) between 2 genes is considered based on the sum of the posterior probabilities of the existence of this relationship (edge) over the time points. In this way, the Gaussian process model is generally considered as nonlinear DBN. Consequently, the developed inference methods, such as the algorithm presented by Aijo and Lähdesmäki [80] and BINGO [53], combine both DBN and GP.

## 4. Inference from time-series of single-cell transcriptomics

**Fig 3:**
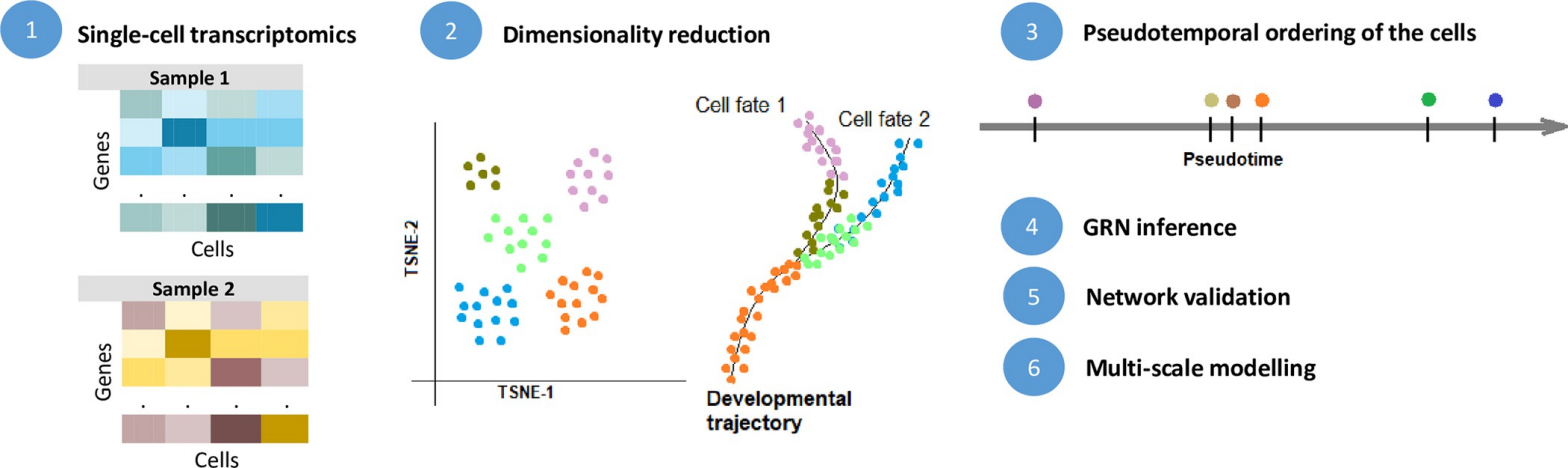
GRN inference from single-cell (pseudo)time-series. Starting from single-cell (pseudo) time-series (1), the inference… workflow follows a similar path as in bulk time-series transcriptomics, except from additional steps of dimensionality reduction and trajectory inference (2), and pseudo-time ordering of the cells (3) when time-resolved experimental measurements are not available. Steps (4–6) follow the same logic as in Fig 2.

For some biological processes, like cell differentiation or phenotypic reprogramming, a higher resolution of the temporal dynamics of gene expression is necessary to identify major phenotypic transitions in complex tissues while characterizing the phenotypic spectrum of individual cells. In this regard, single-cell RNA-sequencing technology enables deeper investigation of the molecular interactions and identification of novel molecular mechanisms that orchestrate biological processes at the single-cell level. Computationally, this technological revolution has led to the development of several algorithms to analyze single-cell RNA-seq and—as a part of it—inference of GRNs (Fig 3). Intuitively, observing genes’ dynamics at single-cell resolution would lead to an increased accuracy in inferring the functional interactions between genes that define the biological process. However, due to the limitations in some of the most widely available single-cell technologies, the heterogeneity and sparsity of single-cell data lead to limitations and challenges for GRN inference methods and put reliability in question.

An important point in using single-cell RNA-seq data for GRN inference is often the lack of time-resolved expression measurements. Instead, many inference methods exploit the multiplicity of RNAseq profiles at one single time point across cells as a proxy for temporal evolution of the phenotype, as is the case for trajectory inference based on pseudo-time ordering of the cells [25], assuming ergodicity of the phenotypes. In this process, the pseudotemporal trajectory is generated by linearly ordering the single-cell profiles from a specific time point based on their transcriptional similarity, thus enabling the identification of gene patterns along the developmental trajectory of continuously ordered cells [86]. Subsequently, to extract discrete time points from pseudotemporal ordering, different techniques can be employed: cell cluster time-point assignment (Slingshot [87], TSCAN [88], or Palantir [89]), partition of pseudotemporal trajectories into discrete time points, or differential gene expression time-point assignment (Monocle [90]). It is important to note, however, that the temporal representation obtained from pseudotemporal analysis in single-cell lacks the equivalence between pseudo-time and real time. In addition, this pseudotemporal representation is better suited in developmental systems, in which cells undergo differentiation processes recognized by the presence of bifurcation points in the pseudotemporal trajectory. This is not always the case, in which occasion trajectory inference may lead to inaccurate results. Nonetheless, this representation helps implement the GRN inference algorithms in modeling the expression levels at a given (pseudo)time-point as a function of gene expression at the previous (pseudo)time-point(s). Accordingly, a subset of the inference methods require specific information about the pseudotemporal ordering of the cells (CellOracle [91], SCODE [20], SINCERITIES [92], SINGE [93], LEAP [94], SCRIBE [95], etc.), having a significant difference in performance when such information is not available. Other methods, like GENIE3 [46], GRNBoost2 [96], or PPCOR [97], do not require a temporal ordering of the cells as input and have relatively good performance when tested on some published curated models [98]. However, the incomplete equivalence between bulk gene expression time courses and pseudo-time time-series implies that these 2 types of inference cannot always be performed by the same tools.

Several benchmarking papers on the performance of single-cell RNA-seq inference methods have been published, facilitating their comparison. We refer the reader to [17,18,21] for an extensive review and comparison of single-cell RNA-seq inference methods, [19,98,99] for some benchmarking libraries, and to [29] for an algorithmic review. In principle, these methods follow a similar classification as in bulk transcriptomics, described in Section 3.2, as many of them were adapted for usage in single-cell from previous existing methods for bulk data. For this reason, here, we simply give a list of some available inference methods (Table 2). We note that there are many more published inference methods, and the list is constantly increasing.

**Table 2:** GRN inference tools from single-cell transcriptomics categorized by their inferring algorithm. The characteristics of the inferred network are indicated as follows: ⊘ undirected; ⊳ directed and unsigned; ▶ directed and signed.

Tool	Method	Language	Reference
⊳ SINCERITIES	Correlation ensemble	R/MATLAB	[92]
⊳ SCIMITAR	Correlation ensemble	Python	[100]
⊳ SCENIC	ODE + Correlariton	R	[25]
⊳ GRNBoost2	Regression	Python	[96]
⊳ CellOracle	Regression	Python	[91]
▶ Pando	Regression	R	[101]
▶ WASABI	Regression + wave propagation	Python	[102]
CARDAMOM, HARISSA	Regression + wave propagation	Python	[103–105]
⊳ PPCOR	Semi-partial correlation	R	[97]
⊳ SINCERA	Correlation	R	[106]
⊘ LEAP	Correlation	R	[94]
⊳ SINGE	Granger causality	MATLAB	[93]
▶ AR1MA1—VBEM	Bayesian Dynamics	MATLAB	[107]
⊳ SCODE	ODE	R/Julia/Ruby	[20]
▶ GRISLI	ODE	MATLAB	[23]
⊳ SCOUP	ODE	C++	[107]
▶ SCRIBE	Mutual Information	Python	[95]
⊘ PIDC	Mutual Information	Julia	[109]
▶ Boolean Pseudo-time	Boolean Model	Python	[110]
▶ InferenceSnapshot	Boolean Model	C++/MATLAB	[111]

For a user, the choice between all the different inference methods will depend on both their overall performance, the type and amount of information they require, and the type of reconstructed network they provide. For example, SCODE, PPCOR, and SINCERITIES infer a directed and signed GRN, which can further be used easily to make dynamical models, if necessary.

## 5. GRN validation

A crucial and essential step in performing GRN inference is validation of the inferred network. Having a validation protocol that allows evaluation of the score of each proposed model is very important in order to choose the optimal inference procedure among the variety of existing algorithms. Despite advances in this direction, evaluating the effectiveness of the inference method remains an open challenge, mostly due to limitations in ground truth/gold standard datasets.

A common approach to evaluate the accuracy of an inference method is to compare the inferred edges to TF–target interactions annotated in public databases, which can be of different kinds, depending on the type of information they contain [30,112]. Some databases will provide information on functional interactions, i.e., evidence that 2 genes/proteins, in this case a TF–target pair, can be related based on any type of link between them, evidenced by gene expression correlation, coevolution of the genes, comention in scientific abstracts, etc. These functional interactions can be found in databases such as String [113], Reactome (the functional interaction network [114], TRRUST [115], or RegNetwork [116], the latest selecting information from around 25 databases. Moreover, there are efforts to map more specific TF–target interactions based on experimental evidence, either collecting results of ChIP-seq experiments that show in which target gene promoters the binding peak of the TF can be found or also combining these experimental results with a crosscheck of the presence of the TF binding motif in the peak regions. Among the databases including these interactions, we mention ChEA [117] or ReMap [118]. Additionally, other databases, generally used when GRN inference is performed on single-cell RNAseq datasets, the validation of inferred interactions is performed on data on the same or similar cell types. Databases providing such information include ENCODE [119], ESCAPE [120], or ChIP-Atlas [121].

However, it is important to note that repositories that report experimental data contain reference data for a reduced number of interactions, limiting the validation to a subset of the inferred network. This implies that the interactions for which the reference data are absent are considered as not existing, raising important questions on the implication of literature biases or the potential to infer novel interactions and regulators. Focusing on prior knowledge for validation might reduce our chances of identifying new regulatory pathways involved in the system under consideration, for which gold standard references with high scores are absent or sparse.

Another possibility for network validation comes from using simulated data, which can be engineered to include several conditions and measurements [14]—yet the extent of coverage of the inferred interactions remains limited to small network size.

Other than validating the inferred network with a gold-standard one, another important point in GNR inference is network comparison between different inference methods, on the same dataset, thus providing an estimate of the “robustness of the inferred network.” Additionally, one may perform this benchmark analysis in order to choose the algorithm/method that best suits a given dataset.

Quantitatively, the algorithm’s performance can be evaluated as for any prediction by 2 metrics typically used in prediction tests: (i) *area under the precision-recall curve* for estimating the performance of a certain algorithm and (ii) *area under the receiver operating characteristics curve* for comparing the GRN inferred by the algorithm against a gold-standard network. Considering every inferred interaction in the GRN as a positive (true—TP, or false—FP) and any missing one as negative (true—TN, or false—FN), the 2 measures are defined as:

1. **area under precision-recall curve (AUPRC):** starting from the inferred weighted GRN, one might start by filtering by the highest values of the weights (*w*_*ij*_∈[0, 1]), for which the precision (the fraction of TP over total predictions) will be high and recall (or sensitivity, the fraction of TP over total true elements) will be low. Adding interactions with lower weights will lead to a decrease of precision, until at recall = 1 (no filter, fully connected network), the precision will represent the fraction of true positives over the total number of edges in the fully connected network. Graphically, this procedure is represented by a precision-recall curve: A good algorithm will have a high precision even when increasing the recall, and an AUPRC close to 1. Generally, this measure is used as a global performance estimator for most of the inference algorithms.
2. **area under the receiver operating characteristic curve (AUROC)**, defined as the ratio between the true positives and false positives, graphically represented with the TP on the *y* axis and the FP on the *x* axis. A good algorithm will have high TP while maintaining low FP values and an AUROC close to 1.

There are numerous other measures of performance that take into account class imbalance more explicitly (F1 value, Matthew’s correlation coefficient, etc.) [122]. In addition, the structural features of inferred networks, such as PageRank, heat diffusion, the shortest path, etc., can be used to compare GRNs inferred from different methods [123], whereas other metrics can be used to define the method performance, such as stability (across simulations, artificially removing measurements), identification of network motifs, and computational time and memory usage [98].

An important question arises when comparing the structure of the same cellular network of 2 different cellular states, e.g., different phenotypes of a certain cell or healthy versus disease conditions. This comparative analysis, referred to as *differential network analysis (DiNA)*, remains a challenge, in parallel with the development of GRN inference methods, especially since different data types other than RNAseq can be included in GRN inference in order to increase the accuracy of the inferred network. For example, using single-cell ATAC-seq, patterns of gene expression can be combined with chromatin accessibility profiles, thus identifying cell subpopulations and groups of cells at different developmental stages [124]. Consequently, several differential network analysis algorithms have been developed, such as DiffRank [125], dcanr R package [126], DiffK [127], DINA [128], to name a few. We refer the reader to [129] for a statistical perspective of DiNA and [130] for a DiNA algorithm comparison. In principle, these algorithms consist of combining the information from 2 main computational tasks: (i) *differential expression analysis*, which estimates the gene expression abundance for each gene in the GRN between the 2 conditions, and (ii) *network expression analysis*, which estimates the importance of each gene in the GRN, based on the topological properties of the network. In this way, DiNA algorithms aim at identifying genes or subnetworks of genes whose expression changes the most across conditions, and they are especially suitable in cases when the changes in the network structure lead to phenotypic changes in the system. Numerous publications have demonstrated the power of this analysis, such as for identifying key TFs involved in cancer, when compared to healthy controls [124].

## 6. Applications

Although inferring regulatory networks is a trending topic and despite the multitude of different algorithms available, GRN inference methods struggle to reach a high performance in real-world studies, on both bulk and single-cell RNA-seq data, as reported in [42,131]. Therefore, their application to biologically relevant datasets remains limited. Recent effort has been focused on combining information from multiple data sources to improve predictions [132,133], where gene expression data are combined with DNA methylation, copy number variation, genome-wide binding data, or CHIP-seq data. As a consequence, many inference methods introduced above combine multiple data sources to complement GRN inference and provide more insightful results on the regulatory processes. For example, in SCENIC [25], results of a GRN analysis are combined with TF binding motif information from RcisTarget to identify a subset of high-confidence interactions. In CellOracle [91] and Pando [101], single-cell RNAseq from a time-series is combined with ATACseq data. These papers illustrate diverse strategies for integrating multiple data sources to enhance predictions and gain a deeper understanding of complex biological systems.

Different applications of inferring GRNs for novel discoveries in biology have been presented in most of the cited works on GRN inference methods, including studies on cancer, cell development, and cell fate decision. In cancer research, special attention has been given to identifying driver genes in cancer progression, and drug resistance. For example, in [85], the authors perform GRN inference from time-series RNAseq in gliomas to build sensitive and resistant networks, found to exhibit significant differences with respect to network topology, local entropy, and gene expression dynamics. Based on the node importance score, they developed a differential regulatory network–based biomarker model to identify the most influential genes for predicting and controlling drug resistance. Going a step further, [134] use time-series of single-cell RNAseq to study epithelial–mesenchymal transition, following a multilayer network approach and linking the intracellular gene regulation to cell–cell communications in ovarian cell lines. Other applications of GRN inference in cancer research focused on identifying putative key regulators and gene modules in PDAC disease progression [135], building cancer cell expression networks in liver hepatocellular carcinoma (LIHC) and bladder urothelial carcinoma (BLCA) [136], analyzing the functional components by extracting subnetworks and investigating the local landscape of prostate cancer genes [137], etc.

Other applications have been focusing on cell differentiation and development [25,103], by integrating different layers of information in GRN inference. For example, in [138], time-series of RNAseq data is combined with ATAC-seq data to derive dynamic gene regulatory networks for human myeloid differentiation, specifically promyelocytes differentiating into macrophages, neutrophils, monocytes, and monocyte-derived macrophages. In Bocci and colleagues [139], single-cell transcriptomics is reinforced with mRNA splicing analysis to identify the key molecular drivers leading to different final states when starting from a common initial state during pancreas endocrinogenesis and epithelial/mesenchymal state transition. In another application, Thorne [140] used public time-series datasets from recount2 database [141] to infer the GRN of neural progenitor cell differentiation. Performing structural analysis on the inferred GRN, they identified key genes like CDON and MCUR1, which were experimentally observed to influence neuronal differentiation. In another approach, Kamimoto and colleagues [91] used single-cell RNAseq and ATACseq datasets to infer cell state–specific GRNs that emerge during the differentiation process of fibroblasts. In this way, analyzing the changes in the GRNs during cell reprogramming or development can help understanding how the TF interactions regulate and define cell identity. In cell fate decision studies, Fleck and colleagues [101] used multiomics datasets, including RNAseq and ATACseq combined with transcription factor binding sites analysis, to infer a GRN describing brain organoid development, leading to the identification of the GLI3 transcription factor as a key regulator of cell fate. Similar studies are presented in [142–144].

In other efforts, data-driven GRNs have been used to build molecular disease maps [145,146], phenotypic characterization of a cell in a given microenvironment [147,148], identifying predictive or prognostic biomarkers [64], performing extensive studies on performance of the available methods on different datasets/conditions [22,124,149], and many more.

## 7. From static to dynamic networks

**Fig 4:**
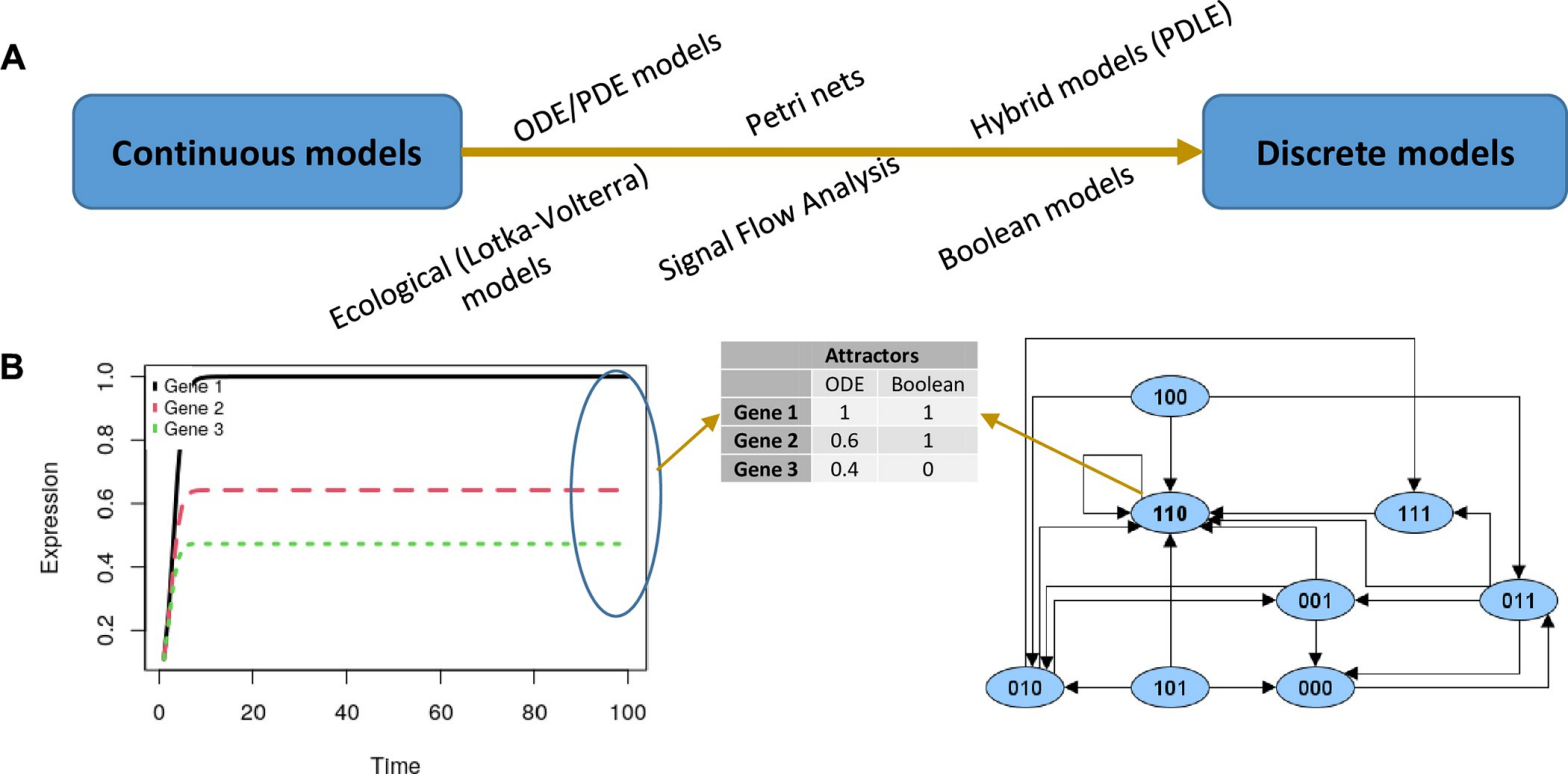
(**A) Dynamical models of gene regulatory networks**, represented as a spectrum of models ranging from continuous to discrete. (**B) Steady states of a system composed of 3 genes:** (left) ODE model, as a system of 3 interacting species; (right) Boolean model, as logic-based interacting entities. The attractors are identified as steady states in the long-term behavior of the system.

The inferred GRNs can serve as a backbone for constructing dynamical models: One can start from a temporal analysis of the system behavior under multiple conditions, describing how the expression of genes in the network changes due to their regulatory interactions. Dynamical models of GRNs cover a spectrum as wide as the GRNs inference methods one. Many dynamical models exist, ranging from continuous quantitative ODE models to discrete logic quantitative models (Fig 4A). In continuous models, the temporal evolution of the state *X*_*i*_ of gene *i*(*i* = 1,2,…,*N*) in the network is given by a continuous ODE function of its regulators. In this way, the regulatory interactions between genes are modeled as chemical reactions, or species interactions in ecological models [150,151]. Beneficially, these models can capture the temporal evolution of the system at the level of individual reactions. However, their usage in dynamical modeling of GRN remains limited because of the detailed and complete mathematical and parametric description they require. On the other side of the spectrum, in discrete models, such as multivariate logic models [152], Petri nets [153], or Boolean models [67,154], a discrete logic of interactions is applied and the temporal evolution of expression of each gene is given by a discrete (and often logic) function of their regulators. Contrary to continuous models, discrete models can be applied with no or considerably fewer parameters and fragmented mechanistic description, making them suitable for large networks. However, the system behavior is only described (semi)quantitatively. Other methods use the network structure to infer the asymptotic behavior of the system, i.e., identifying only the steady states of the system, considering only the static GRN and a set of initial conditions, without requiring a dynamical description [39,40].

For a modeler, the choice of the modeling method is going to depend on the type of questions being asked on the system, the type of available description of the system dynamics (quantitative or qualitative), the type and amount of available data, prior knowledge, etc. Whichever the dynamical model used, the main goal is to identify the phenotypic changes of a cell in response to certain extracellular environmental conditions, to drugs, to cellular interactions in the microenvironment, or in experimental knockout/overexpression experiments. Graphically, these phenotypic changes can be identified as the system’s steady states or attractors (fixed points or limit cycles) (Fig 4B) [155], defined as a state (a vector of expression values of each gene), which remains unvaried even in the presence of perturbations. Usually, a GRN can have multiple attractors, representing the possible phenotypes or cell states that can be reached when starting from the given initial conditions. In this case, further analysis must be performed on the attractors, to study their stability, their biological relevance, or—additionally—their categorization into known phenotypes.

Particularly in Boolean models, similar to using time-series of expression data for GRN inference, the temporal information in the changes of the genes’ expression is also used to infer the Boolean rules that govern these changes [115,156–161], which can be then studied using several available tools [162]. More recently, patient-specific Boolean models have been developed to suggest targeted therapy to patients based on their ‘omics profile [163]. Importantly, these intracellular models can be further integrated with other cell population models, like agent-based models or metabolic models, thus providing a multilevel description of the system dynamics, including processes like cell motility, cytokine diffusion, tissue expansion, spatial organization, etc. [164–167]. This combined approach enables addressing more complex tasks, such as drug design, or prediction of therapy response, from the cell to the tissue scale.

## 8. Discussion, open challenges

In this review, we give a basic introduction to GRNs, their topological characteristics and, most importantly, we describe the main GRN inference methods. Our aim is to give life scientists an overview of how to use the abstract concept of GRNs to investigate complex cellular molecular interactions more thoroughly and identify how specific interactions determine cells’ behavior and response to the environment. With the increasing abundance of transcriptomics data, data-derived GRNs have the potential to capture novel gene interactions and help to expand our knowledge of important molecular pathways. Despite the increasing interest in developing highly performing GRN inference methods, and this field of research having been active for more than 20 years, often the application of even the best performing methods in real-world studies raises questions about their reliability [131]. Purely data-driven GRN inference remains an open challenge, especially in single-cell RNAseq. Recent promising research addresses the task of network inference in the presence of incomplete multiomics datasets, resulting in the development of advanced computational methods [158]. We speculate that perhaps the information provided by transcriptomics data, even in single cell, is not sufficient to cover the complex cellular processes giving rise to phenotypes and that the inherited concept that coexpression patterns might reveal putative gene interactions might not be universally applicable. For example, due to posttranscriptional and posttranslation processes, a highly expressed mRNA may not lead to a highly abundant functional protein, and vice versa—a synthesized protein does not necessarily possess the necessary activation state or conformation or localization to affect its downstream targets. Therefore, there is a necessity to produce multiple types of ‘omics data, consider different biological entities and levels of regulation, and the challenge in performing GNR inference will consist of integrating the data effectively.

Additional limitations in the performance of GRN inference methods can come from other—often ignored—sources. For example, gathering experimental samples from various patients can suppress the variability, since patients have individual histories, immune system, or genetic profiles. Experimental protocols can present restrictions as well, such as limited measurements of expression (transcription, degradation, sequencing capture, etc.), heterogeneity in bulk datasets, or incompleteness in single-cell RNAseq transcriptomics.

Another challenge in data-driven GRN inference is result interpretability and the difficulty in dealing with the high complexity of the resulting networks increasing with their size. When considering cell state transitions (like differentiation or polarization) in a multicellular system, we can imagine that the interactions between TFs and their target genes can be cell type specific, potentially requiring different GRNs. However, all of the inference methods produce a single GRN, thus failing to provide the dynamics of the underlying mechanisms during cell state transition captured by the time-series. One possibility in dealing with this challenge is to infer stage-specific GRNs, thus having a time-evolving GRN that can help understanding how the involvement and interactions of specific genes/TFs lead to cell state transition. However, studying and understanding time-evolving GRNs remains an unexplored field of high complexity. Additionally, tracking specific regulatory pathways or interactions rapidly becomes a real challenge when dealing with large networks consisting of thousands of genes. We assume that topological network analysis might help in reducing the network size by selecting the most important nodes (by their centrality measures), although there is still a gap in our understanding between the structural and dynamical properties of the network [13,168–171]. Centrality measures can be sensitive to noisy or incomplete data that affect the structure of the inferred network. While such centrality measures are widely used to study the structural properties of many types of networks (social networks, communication networks, etc.), they might fail to capture the complex nonlinear interactions of gene regulation that are key in biological regulatory networks. We speculate that the existing challenges of data-driven inference methods discussed above are tightly intertwined with the limitations displayed by centrality measures derived from structural analysis of GRNs. An alternative could be the combination of multiple genes into functional units (gene modules, pathways) that could be nodes of a smaller GRN, thus reducing the dimensionality of the problem. This would still require quite arbitrary choices about how to group genes, but identifying functional modules could be achieved [172].

Using advanced computational methods, recent efforts have helped tackle the challenge of filling the gap between static and dynamic properties of GRNs. For example, in their work, Marazzi and colleagues [40] developed a method to combine several layers of information: gene expression data, the static representation of GRNs, the concept of system’s asymptotic behavior, and machine learning methods, to identify cell fate reprogramming targets. In another approach, Meena and colleagues [173] use the information on network topology to apply the linear approximation of dynamical Jacobian ensemble, thus qualitatively investigating and classifying the system’s asymptotic behavior around the fixed points.

One of the most difficult challenges in performing data-driven GRN inference is validating the results and estimating the method performance in real case studies. Benchmarking the inference methods with simulated datasets from a prior knowledge network (PKN) and a small list of genes can be an efficient way to estimate the methods’ performance. However, this is far from real biological systems, in which the actual regulatory mechanisms are mostly unknown and—in some cases—experimentally unexplored. Unfortunately, using public databases (usually built on bulk-RNAseq) for validating the inferred GRNs can be limited to small-size GRNs and narrowed around the highly studied regulatory pathways. Consequently, many approaches can lead to biased conclusions and a real difficulty in identifying novel regulatory pathways that might play an important role in the system under study.

We believe that many of these challenges will be addressed in integrative inference methods to be developed in the future. Despite the considerable improvements and the rapid growth in numbers of GRN inference methods, this remains a relatively new and highly complex field. Feeding the methods with different types of ‘omics data and prior knowledge, GRN inference can help discover the importance of—yet uncharacterized—pathways, biological components, and interactions, thus further increasing our knowledge that will serve as a starting point for even more complete models, in a positive feedback loop.
